# Patient Expectations in High‐Risk Abdominal Surgery for Cancer

**DOI:** 10.1111/hex.70764

**Published:** 2026-07-12

**Authors:** Kimberly E. Kopecky, Jacquelyn E. F. Speer, Janet Julson, Alizeh Abbas, Olivia Monton, Fabian M. Johnston, J. Nicholas Odom

**Affiliations:** ^1^ Department of Surgery University of Alabama at Birmingham Birmingham Alabama USA; ^2^ Heersink School of Medicine University of Alabama at Birmingham Birmingham Alabama USA; ^3^ Department of Surgery McMaster University Hamilton Ontario Canada; ^4^ Department of Epidemiology Johns Hopkins Bloomberg School of Public Health Baltimore Maryland USA; ^5^ Department of Surgery Wake Forest University School of Medicine Winston‐Salem North Carolina USA; ^6^ School of Nursing University of Alabama at Birmingham Birmingham Alabama USA

**Keywords:** expectations, patient perspective, surgery

## Abstract

**Background:**

Despite the central role of expectations in informed consent and surgical shared decision‐making, patient expectations frequently remain unelicited or underexplored and are often assumed rather than explicitly addressed.

**Objective:**

To explore how patients form expectations about high‐risk abdominal surgery for cancer and how preoperative expectations influence the experience of surgical recovery and life after surgery.

**Design:**

Qualitative study using semi‐structured interviews informed by cognitive task analysis and analysed using an abductive thematic framework.

**Setting and Participants:**

Adults (*n* = 34) at two U.S. academic hospitals who were either scheduled to undergo, or had recently undergone, high‐risk abdominal surgery for cancer; interviews were conducted preoperatively (52.9%) and postoperatively (47.1%).

**Results:**

Analysis of the interviews revealed three overarching themes characterising how patients formed, understood, and experienced expectations around high‐risk cancer surgery. Theme 1: Origin of Expectations‐ Expectation development was dynamic, context‐dependent, and shaped by multiple information sources. Theme 2: The Complexity of Expectations‐ Patients varied widely in how much they wanted to know; expectations were often internally inconsistent and frequently conflated with hopes. Theme 3: Contrasting Anticipated and Actual Recovery After Surgery‐ Postoperative experiences commonly diverged from preoperative expectations, and many patients expressed uncertainty regarding recovery and prognosis, even in medically uncomplicated recoveries.

**Discussion:**

The wide variation and inconsistency in patient expectations reflect both the complexity of how patients prepare for high‐risk abdominal cancer surgery and their need to navigate uncertainty in ways that align with their individual preferences, values, and tolerance for information. Mismatches between anticipated and actual postoperative experiences underscore the need for structured, patient‐centred communication strategies that support realistic preparation for surgical recovery.

**Conclusion:**

This study provides insight into how patients with cancer form expectations regarding high‐risk abdominal surgery and how these expectations shape preparation for the experience of surgical recovery. Expectations were often incomplete, internally inconsistent, and difficult for patients to articulate, contributing to gaps between anticipated and actual recovery. These findings highlight the need for intentional, patient‐centred approaches to elicit, clarify, and better align patient and clinician expectations in surgical oncology. Future work should evaluate intervention strategies to support this goal.

**Patient or Public Contribution:**

Patients participated in in‐depth interviews that form the basis of this study. Caregiver interviews were conducted in parallel as part of a related study but were not included in the present analysis. Patients and/or caregivers were not formally involved in the design of the study or in the analysis of the qualitative data.

## 1 Introduction

Little is known about how patients form expectations about high‐risk cancer surgery (≥ 1% operative mortality) [1], or how expectations influence their decision‐making and the overall surgical experience [2, 3]. Despite the unspoken central role of expectations in informed consent and shared decision‐making [4, 5], expectations often remain unelicited and underexplored and are frequently assumed rather than purposefully addressed in clinical encounters [6, 7, 8]. In fact, current models of shared decision‐making fail to explicitly elicit patient and caregiver expectations [4, 9, 10, 11, 12, 13], potentially contributing to a mismatch between what patients anticipate surgery will be like and the actual experience.

Understanding patient expectations in the context of high‐risk surgery is essential, given that expectations shape how patients prepare for surgery and life after surgery, evaluate satisfaction with outcomes, and navigate recovery [14, 15, 16, 17]. When patient expectations remain unclear, unexamined, or unrealistic, surgeons may struggle to provide value‐concordant treatment recommendations, as patients’ decisions may be driven by assumptions about prognosis or recovery that do not reflect clinical reality [18, 19, 20, 21, 22]. Moreover, research suggests that mismatches between expectations and reality may leave patients feeling anxious, fearful, stressed, disappointed, angry, and resentful during and after surgical recovery.

Given the centrality of expectations in surgical decision‐making, the aim of this study was to explore how surgical expectations are formed, what influences them, and how they shape preparation for and the experience of recovery among patients who were scheduled for or had recently undergone high‐risk abdominal surgery for cancer.

## 2 Materials and Methods

### 2.1 Study Design

This was a qualitative study conducted across two academic centres using a cognitive task analysis (CTA) interviewing approach. Two authors with expertise in qualitative methods (K.K., J.N.O.) and surgical oncology (K.K.) developed a semi‐structured interview guide following established CTA methods, which was reviewed by an additional study team member (O.M.), given her content and methodological expertise in qualitative research, surgery, and patient‐centred communication [23, 24, 25, 26, 27, 28]. The CTA approach was selected to elicit detailed insights into how patients cognitively process and construct expectations in complex decision‐making contexts, including how they make sense of uncertainty and anticipate recovery. Preoperative interviews focused on prospective expectation formation, whereas postoperative interviews used CTA techniques to guide participants back to the time of diagnosis and preoperative decision‐making, allowing them to reflect on their thoughts, understanding, and expectations at that time. Accordingly, the early portions of interviews were consistent across groups. Each question in the interview guide was matched to a CTA construct to ensure alignment with CTA principles. A target sample size of 30 participants was set based on feasibility (weekly volume of high‐risk [1] oncologic surgery at two participating academic hospitals) and evidence regarding the number of interviews needed to achieve thematic saturation [29]. This study was reviewed by the Institutional Review Boards at both participating institutions and was determined to be exempt due to minimal risk and the use of voluntary, interview‐based data collection. Additionally, the manuscript was reviewed for alignment with the Consolidated Criteria for Reporting Qualitative Research (COREQ) to enhance reporting transparency and completeness [30].

### 2.2 Eligibility

Surgical patients were eligible to participate if they were at least 18 years of age, English‐speaking, and were scheduled for or had recently undergone high‐risk abdominal surgery for cancer at a participating site (The Johns Hopkins Hospital in Baltimore, MD or the University of Alabama at Birmingham in Birmingham, AL). High‐risk surgery was defined as procedures associated with at least 1% operative mortality [1]. Exclusion criteria included interviewer‐identified limitations in the participant's ability to meaningfully participate in the interview (i.e., cognitive impairment, dementia).

### 2.3 Screening and Recruitment

Participants were purposively sampled to include individuals interviewed both preoperatively and postoperatively to capture a range of perspectives and experiences. Eligible participants were identified by the first author (K.K., female surgical oncologist) by reviewing the operative schedule for upcoming or recently completed high‐risk abdominal surgeries. Patients were contacted sequentially based on predefined inclusion and exclusion criteria, by telephone, using information available in the electronic health record (EHR). The study protocol outlined recruitment of preoperative participants within 3 weeks of their scheduled surgery date and postoperative participants within 4 months following surgery. Up to three contact attempts were made at varying times of day; individuals who did not respond were considered non‐responders and were not further pursued. During initial contact, an overview of the study was provided, the voluntary nature of participation was emphasised, and participants were informed that their decision to participate or decline would not affect their clinical care. While a formal recruitment rate was not prospectively tracked, we estimate that more than 50% of approached patients agreed to participate. Interested individuals completed verbal consent and were scheduled for a telephone or videoconference interview at their convenience. Participants were assigned unique study identifiers at the time of enrolment; simplified identifiers (P1–P34) are used in the text for readability. Notably, participants completed either a preoperative or postoperative interview; no participants were interviewed at both time points.

### 2.4 Data Collection

Sociodemographic and clinical information were captured from the EHR following enrolment. The first author (K.K.), a surgical oncologist with training in qualitative methods, conducted all qualitative interviews between November 2023 and December 2024. Over the first 12 interviews, the semi‐structured interview guide was iteratively refined to incorporate additional in‐depth probing questions related to emerging themes. Core domains remained consistent across all interviews, and modifications were limited to enhancing exploration of concepts identified during early data collection. Participant interviews were audio‐recorded using a HIPAA‐compliant Zoom account and transcribed verbatim by an external transcription service.

### 2.5 Data Analysis

Transcripts were uploaded into NVivo 15 for qualitative analysis. The initial codebook was drafted by the first author (K.K.). Analytic memoing was used to document preliminary impressions and capture the early emergence of potential codes and themes. An abductive analytic approach [31, 32] was used to integrate theory‐informed codes derived from the interview guide with inductive codes emerging from the data, allowing for both structured and exploratory analyses of patient expectations. The codebook was iteratively refined with input from additional team members, who reviewed transcripts, proposed new codes, and helped clarify definitions and structure. Four coders (K.K., J.S., J.J. and A.A.) independently coded the data, with each transcript independently coded by two coders to ensure diverse perspectives and consistency in interpretation. Discrepancies were resolved through discussion and consensus. Codes were grouped into broader thematic categories, and subgroup analysis was performed for postoperative interviews. After further refinement, all authors reached consensus on the final themes.

## 3 Results

Of the thirty‐four participants interviewed, 18 (53%) were recruited from The Johns Hopkins Hospital, and 16 (47%) were recruited from the University of Alabama at Birmingham. Interviews were conducted both preoperatively (52.9%) and postoperatively (47.1%). The mean number of days before surgery for preoperative interviews was 12.5 (range 5–19), and the mean number of days after surgery for postoperative interviews was 49.8 (range 21–116). See Table 1 and Supplementary Table A in the Appendix for detailed participant characteristics.

**Table 1:** Participant characteristics.

	N = 34
Institution	
The Johns Hopkins Hospital	18 (53%)
University of Alabama at Birmingham	16 (47%)
Interview type	
Preoperative	18 (53%)
Postoperative	16 (47%)
Age (years)	58.5 (31–85)
Sex	
Male	16 (47%)
Female	18 (53%)
Race and Ethnicity	
White	19 (56%)
Black/African American	9 (26%)
Asian American	3 (9%)
African American White	1 (3%)
Declined	2 (6%)
Marital Status	
Married	19 (56%)
Divorced	6 (18%)
Widowed	5 (15%)
Single	4 (12%)
Religious affiliation	
Reported	24 (71%)
Not reported	10 (29%)
Diagnosis	
Pancreatic cancer	12 (35%)
Lower gastrointestinal cancer	9 (26%)
Liver/biliary or gallbladder cancers	6 (18%)
Neuroendocrine tumour	3 (9%)
Retroperitoneal sarcoma	2 (6%)
Gastrointestinal stromal tumour	1 (3%)
Surgical procedure^a^	
Pancreaticoduodenectomy	9 (26%)
Hepatectomy/liver ablation/radical cholecystectomy	7 (21%)
Cytoreductive surgery with hyperthermic intraperitoneal chemotherapy	6 (18%)
Distal pancreatectomy/splenectomy	5 (15%)
Colonic resection/low anterior resection/abdominoperineal resection	3 (9%)
Gastric or small bowel resection	2 (6%)
Procedure type	
Open	26 (76%)
Robotic	8 (24%)

^a^ Surgical procedures are classified according to the main procedure type.

**Figure 1:**
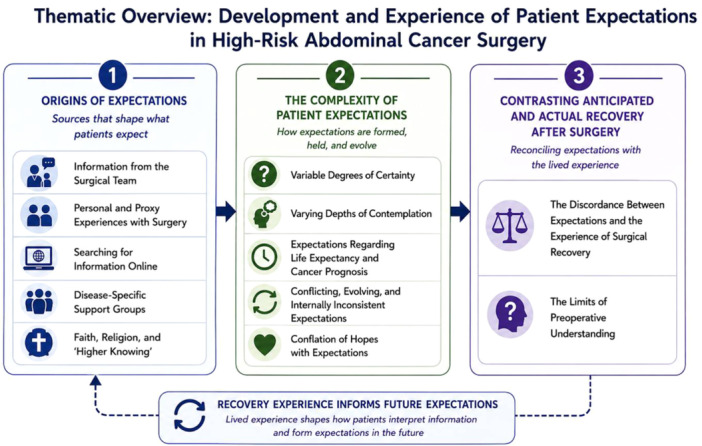
Development and experience of patient expectations in high‐risk abdominal cancer surgery. This graphic summarises the major themes identified through qualitative interviews with patients undergoing high‐risk abdominal surgery for cancer. Patient expectations were shaped by multiple sources, including information from the surgical team, personal and proxy experiences, online information seeking, support groups, and faith‐based beliefs. Expectations were further characterised by variable certainty, differing levels of contemplation, evolving and internally inconsistent beliefs, and frequent conflation of hopes with expectations. Patients often experienced discordance between anticipated and actual postoperative recovery, highlighting the limits of preoperative understanding. The iterative feedback loop illustrates how lived postoperative experiences may subsequently influence how patients seek, interpret, and form future expectations.

Analysis of the interviews revealed three overarching themes characterising how patients formed, understood, and experienced expectations around high‐risk cancer surgery, see Figure 1. Themes 1 and 2 were broadly consistent across preoperative and postoperative participants, whereas Theme 3 (anticipated vs. actual experiences of postoperative recovery) was derived from postoperative participants and reflects their lived experience of recovery.

### 3.1 Theme 1: Origins of Expectations Among Cancer Patients Considering High‐Risk Surgery

Patients drew on a wide range of sources when forming expectations, including information provided by the surgical team, prior personal and indirect experiences with cancer, online information that was sought proactively, disease‐specific support groups, and, for some, faith or an internal sense of knowing. See Table 2.

**Table 2:** Origins of expectations among cancer patients considering surgery. How they knew what they knew.

Information from the surgical team	Because of what [the surgeon] has told me, I expect to have some pain and discomfort, more discomfort. (P32)
	They explained it very well. They even went through little graphics for how they was gonna proceed with it. (P19)
	Like I said, [the surgeon] did tell us it was gonna take up to 12 weeks for the healin’, and we're prepared. (P26)
Personal and proxy experiences with surgery	From my last few surgeries I knew that it just took time and patience and eventually I'll be back to normal. (P13)
	There was a lot of pain management involved with the colon surgery. I know this is gonna be very similar. (P34)
	We didn't expect it the first time, but because it happened the first time, we were prepared mentally for it to happen the second time. (P27)
Searching for information online	I did my research again through Facebook and Google and all the researches *[sic]* that are out there. (P1)
	I went online [and] I was able to see a diagram of what they were going to do and that kind of thing. (P2)
	I think probably I don't ask a lot of questions from the doctors because I think I have a lot of the answers. I've already done a lot of the research myself. (P6)
Disease‐specific support groups	I was fairly aware of what was going on through [the Facebook support group]. (P6)
	I found a group for pancreatic survivors, and I had no clue that there are actually survivors. (P1)
	You get some good advice, but 90 percent of the time, you have to take everything with a grain of salt. (P29)
	It can be really depressing, but you also can find so much information. (P6)
Faith, religion, and ‘higher knowing’	I'm not a worrier. I put it in the Lord's hands and let him deal with it. I just do what I'm supposed to do. (P34)
	I believe that God will be there for me, and it makes a huge difference. (P24)
	I don't know necessarily getting through it but knowing that whatever happened, it was going to be fine. (P22)
	I just knew that I was going to be all right from this for some reason. I would tell people I had to go in and they'd tell me they were going to pray for me, and I just thought everything was going to be good. (P4)

#### 3.1.1 Information From the Surgical Team

Expectations were shaped by the content, tone, and clarity of information provided by the surgeon and the surgical care team. Patients understood that surgery was '*hefty'*, '*serious'*, '*big'*, '*major', 'invasive',* and '*severe*' based on how surgeons described the operation, including the extent of the procedure. A 79‐year‐old female with pancreatic cancer described, '*The pancreas is behind your liver, your gallbladder, all the other things… they have to go and loosen them up and then operate on the pancreas, and then tie them all back together and put them in place. So I know it was a serious operation'* (P4). At other times, the way surgeons spoke about less complex procedures helped patients calibrate their expectations regarding overall risk and anticipated postoperative needs. A 77‐year‐old female patient with a gastrointestinal stromal tumour (GIST) noted, *'He looked at his schedule and he said, “I've got four surgeries scheduled that day, I'll just tuck you in at the beginning. We'll do you, and then I can move on to the others”*' (P32). Additional cues about the seriousness of the operation included the anticipated operative time and the need for a planned postoperative ICU admission. As another 41‐year‐old female with appendiceal goblet cell recalled, '*I think that when [the handout] talked about going into the ICU right after surgery, that made me realize like, “Oh, this is a big deal”*' (P13).

Some, but not all, patients received information regarding postoperative pain, dietary needs, and mobility considerations. When this information was given, patients found it helpful. Most commonly, patients were expected to intuit what recovery would entail, relying on past experiences, assumptions, or observations of others to fill in the gaps. Many patients were told that they would have pain, and were also given guidance on the number of days they were anticipated to be in the hospital. When probed for more details beyond this, several interviewees, including this 64‐year‐old male patient with pancreatic cancer disclosed *'I was never really told what to expect, so I just don't know'* (P2). Others echoed this uncertainty, with another 54‐year‐old male patient noting, *'I didn't know what would happen, to be quite honest with you'* (P3). When specifically asked about postoperative dietary expectations and physical needs, some patients offered speculative answers or avoided answering the question at all. For example, when a 41‐year‐old female with pancreatic ductal adenocarcinoma (PDAC) was asked about her expectations regarding appetite in the postoperative setting, she responded, *'I'm assuming that I'll be very hungry'* (P7). When another 40‐year‐old female was asked about how prior surgical experiences impacted her expectations of recovery following pancreaticoduodenectomy, the patient exclusively shared information about the institution that she had found online (P1). In the absence of clear guidance regarding postoperative recovery and life after surgery, many patients that were interested in learning more drew on additional sources of knowledge were available to them.

#### 3.1.2 Personal and Proxy Experiences with Surgery

Patients drew on previous surgical experiences and the experiences of close friends and family members to inform expectations for their own cancer‐related surgery. Some patients felt that the process and logistics of planning for surgery were similar to their prior surgical experiences, while the majority of participants noted that because the surgery was being performed for cancer it felt more serious. A 77‐year‐old female with GIST noted '*The process does not feel different. The cause feels a lot different*' (P32). Similarly, a 42‐year‐old female patient with anal cancer shared, *'It feels like five times bigger than those other surgeries [cholecystectomy, appendectomy, c‐section]'* (P29) and a 54‐year‐old female with colorectal cancer metastatic to the liver reflected, '*I look at this one as being much more serious… [cancer] is a very serious matter'* (P16).

For some patients, their experience recovering from prior surgery helped them anticipate what recovery from abdominal cancer surgery might involve. This included specifics such as waking up from surgery with a nasogastric tube, surgical drains, and a urinary catheter, as well as the routines of being in the hospital and convalescence at home. The 54‐year‐old male with liposarcoma had previously experienced a postoperative infection following elective surgery shared, *'On one hand, I had an infection following one surgery, so I know that things can become difficult. On the other hand, most of my surgeries have been fairly, for lack of better words, easy, with a quick recovery'* (P3). Similarly, a 41‐year‐old female patient with metastatic colorectal cancer to the peritoneal cavity explained that she felt reassured by a previous experience of achieving normalcy after surgery, *'I think it's helpful because I know I was taken care of. I know I wasn't allowed to sit in pain. I know that I can recover and that my life can get back to some sense of normalcy'* (P6).

Experiences supporting family or friends who had undergone cancer surgery also informed patients’ beliefs about their own expected outcomes and recovery. The 41‐year‐old female with PDAC explained that her mother had undergone the same procedure that she was preparing for, and explained, *'I have a unique situation because my mother passed away from the same exact disease 12 years ago. So, I've kind of been through the whole process already [and] when I was diagnosed, I knew what it meant… that I would be getting surgery'* (P7). Other patients referenced extended family members with ostomies or drew on conversations with friends to whom they disclosed their diagnosis. Most often, these discussions offered reassurance that someone else had been through something similar, but they rarely provided concrete details about the experience of surgery. These secondhand experiences provided patients with a sense of support and confidence in their own recovery, though the depth, accuracy, and applicability of these impressions varied widely.

#### 3.1.3 Searching for Information Online

Online searches, institutional cancer‐specific webpages, and open‐access scientific publications and abstracts available on the internet contributed to expectation formation amongst those who sought out this information. A 40‐year‐old female with PDAC who was scheduled to undergo pancreaticoduodenectomy shared, *'I've done a lot of research about it… through Facebook and Google and all the researches [sic] that is out there'* (P1). Other patients admitted to watching posted surgeries on YouTube. Some patients noted that because they had read a bit about surgery on the internet, they felt less strongly about asking their surgeons questions. A 41‐year‐old female patient with colon cancer disclosed, *'When I met with [the surgeon], what he said totally aligned with what I had already researched and as such, I didn't really ask a ton of questions'* (P6). A 36‐year‐old male patient noted that reading information online *'mentally prepared me for the whole thing, because I wanted to know exactly what was going to happen…[it] helped me cope with the reality of the whole situation'* (P14). Some patients found online graphics and visuals helpful for understanding the surgical procedure itself, but admitted that this information did little to prepare them for what to expect during recovery or life after surgery, while other participants did not have interest in reading information online and felt more comfortable talking with the healthcare team directly. As a 59‐year‐old‐female patient with PDAC put it, *'if you look on the internet, it might tell me things I don't want to know…so I just follow what the doctors tell me'* (P18).

#### 3.1.4 Disease‐Specific Support Groups

Interactions with others in cancer or illness‐related support groups provided real‐world insights that shaped expectations. Patients found support groups most commonly by performing Google searches and exploring social media platforms such as Facebook and Reddit. Having access to a community of people navigating a similar diagnosis or who had already undergone surgery was helpful for some, as it exposed them to a range of personal experiences and potential postoperative outcomes. The 41‐year‐old female patient with metastatic colon cancer described, *'People share both the whole spectrum of, “Oh, I was out of the hospital in 5 days,” I sort of knew the gamut of outcomes'* (P6). While a subset of patients actively contributed to these online communities, many engaged primarily as observers, reading the accounts, questions, and posts shared by others. For example, the 40‐year‐old female with PDAC explained, *'I don't really post my personal stuff because I always feel like I'm going to jinx myself or something. I always read and just read through the comments, and then you find other people that are similar to me, and so I look at how they experience things'* (P1). Less commonly, patients mentioned turning to non‐social media‐based advocacy and support networks like PanCAN [33] for additional information and guidance.

#### 3.1.5 Faith, Religion, and ‘Higher Knowing’

Faith, spiritual frameworks, or a sense of higher ‘knowing’ influenced how patients anticipated their surgical journey and shaped their expectations. For some, religious beliefs provided a clear anchor for what they expected, the 54‐year‐old female with colon cancer metastasised to the liver shared, *'That's my prayer and that's my hope. That's what I believe will happen. That's my expectation'* (P16). Spiritual or religious faith helped many participants feel grounded amid uncertainty and fostered a sense of reassurance that everything would be okay, a 79‐year‐old female with PDAC noted, *'I just knew that I was going to be all right from this for some reason'* (P4). Others described a more intuitive or non‐religious sense of knowing, an inner certainty or trust, that guided their expectations.

### 3.2 Theme 2: The Complexity of Patient Expectations

Patients experienced a wide range of expectations about cancer surgery, reflecting differing levels of certainty, cognitive engagement, anticipated future well‐being and internal consistency. Variations in expectations both across patients and *within* the same patient reflected highly dynamic processes shaped by emotion, information delivery, life experience, and individualised coping strategies.

#### 3.2.1 Variable Degrees of Certainty

Patients expressed varying degrees of certainty regarding their expectations for postoperative recovery and life after surgery. Some patients conveyed an optimistic outlook about postoperative recovery (i.e., quick recovery), the intraoperative events (i.e., uneventful surgery), and postoperative pathologic findings (i.e., negative lymph nodes). Others articulated clear and confident expectations of returning to a previous or even improved state of health and functioning. A 40‐year‐old female with PDAC shared, *'my expectation is once he's in there and he says it's good to go, that the tumour will be removed, that there's no issues with my arteries or veins …that my lymph nodes come back all clear, that when I wake up and he tells me clear margins everywhere'* (P1). Some patients had a vague understanding of surgical recovery and acknowledged uncertainty about how things would unfold. For some, uncertainty generated distress and fear, while others seemed to be more ambivalent and were not worried or bothered by it. These differences reflected how patients internally negotiated hope, fear, and realism when imagining life after surgery. See additional participant quotes in Tables 3 and 4.

**Table 3:** Degrees of certainty related to expectations among cancer patients considering surgery. Patients reported a wide range of certainty related to differing aspects of the postoperative experience.

	High uncertainty	In between	High certainty
Expectations regarding the physical aspects of surgical recovery	I don't really have any expectations. I'm just going to go with the flow. (P16)	The first few days, I may eat liquid only, and slowly increase. (P9)	I'm expecting just full recovery, being better than I was… when all this started. Yeah, being back to normal. (P11)
	I'm kind of expecting to be in the hospital for a few days, I guess. (P12)	I would guess I'm gonna need help with things for probably a couple of weeks or so. (P34)	I don't expect that any lifestyle choices or that my lifestyle will be affected by this surgery. (P32)
	I'm gonna need some help getting out of bed maybe? I don't know. (P24)	I'm particularly curious about how much pain I'm going to be in when I wake up. (P12)	I expect it to be pleasant. He said wonderful. He said up to 4 days. (P32)
	To be honest, I don't know, I don't have any really expectations. (P11)	How my guts are waking up and how this is going to play, I don't know yet. (P1)	It'll definitely change the way I eat. It will definitely change my exercise habits. (P16)
	I was never really told what to expect. (P2)	I think it'll change my quality of life. (P16)	I wake up and he tells me clear margins… no infections, of course, and that I can pretty much go up and eat normal. (P1)
	I don't know what to expect in terms of how long I'll be uncomfortable, how long I'll be in pain, what kinds of effects the surgery would have. (P23)	It seems like it can be normal. Of course, my diet would probably have to change, which is okay. (P11)	I'll have some compromise in my functionality and mobility, that my recovery period that is back to my ability to engage in normal life activities will be approximately 2 to 3 weeks. (P32)
	I really haven't—I guess I really haven't thought about my expectations about what to expect in the hospital. I'm terrified. I'm terrified to even get to the hospital. (P29)	I don't see it being easy. I mean, I'm hopeful that there's no issues outside of the standard issues, but I know that it's not going to be an easy recovery. (P29)	I expect to have some pain and discomfort, more discomfort. I expect to have some compromise in my mobility. Those are the three things that occurred to me. (P32)
	It's unknown, in terms of the side effects and whatnot, of course. (P23)	I would hope by next summer, the pain has gone for the most part. (P7)	I don't expect any compromises in my abilities. (P32)
Expectations regarding survival/cure and prognosis	The worry will always be there that it will come back or it could come back. (P1)	I will be always anxious for the scans and stuff, but hopefully I'll be living till I'm 77 or so. (P1)	I don't anticipate [the cancer] coming back. (P16)
	I do hope I will be as normal as possible. (P1)	I know that it's not a guarantee of cure. (P6)	I'm expecting to live another 20 years. (P23)

**Table 4:** Conflation of hopes with expectations. Patients frequently conflated hopes with expectations.

	Interviewer question	Participant response
I.	What are your expectations about the outcome of surgery?	My hope is that it's curative. That's really my main hope. (P6)
		I'm expecting one and done… cut it, barbecue it. Never come back. That's my prayer and that's my hope. (P16)
		My hope is that the outcome is that the tumour was just where they thought it was, and it's encapsulated and not touching any blood vessels and hasn't spread to any lymph nodes, and that they can just remove it and everything's fine. (P7)
II.	How do you think that you're going to feel after you've been home for a few weeks?	Well, I hope that I will be fine and… that after 5, 6 weeks, I may be able to go back to work. (P1)
		I mean, a few weeks‐‐ I'm hoping by week 4, I'm starting to feel a little bit more like myself. I'm walking a lot more. I'm kind of up and about and the pain is dull. (P7)
		Well, I hope I will be, I'm hoping I will be okay. So yeah, I don't know. To be honest, I don't know. (P11)
		I'm hoping I'd feel like the way I did the day before surgery, in that I'm not in any pain, I'm not in any discomfort related to the cancer. (P23)
		I'm hopeful to be mobile and able to walk around, go do something out of the house. (P6)
		Hopefully eating a little bit more each day or finding what works for me by that point at the very least. (P7)
III.	What are you expecting it to be like when you wake up from surgery and when you're in the hospital recovering?	I hope when I wake up I am not in a whole bunch of pain. (P11)
		I hope it's going to go good, and that I don't have to throw up a lot, and that I don't feel nauseous and definitely no pain. (P1)
		I hope he will be at my bed and tell me, 'Everything went fine. We were able to do the surgery and I'll touch base with you. Everything is fine. Don't worry about it'. (P1)
		I'm hopeful that there's no issues outside of the standard issues, but I know that it's not going to be an easy recovery and I'm incredibly anxious about that. (P7)
IV.	How do you think about the differences between expectations and hopes?	My hope and my expectations are the same thing… I'm expecting a miracle. (P11)
		No, it's the same. My hope is the same as my expectations, and I consider that to be a blessing. (P32)
		So 95% of the time, my expectation falls in that hopeful place, and I think that it's important to try to align yourself with that. But there's that 5% where I'm like, 'Okay, I have pancreatic cancer and I know all the statistics', and I know how this can go a million different ways. (P7)

#### 3.2.2 Varying Depths of Contemplation

Patients demonstrated differing levels of reflection and contemplation when considering recovery and life after surgery. For some, their expectations were shaped by deep introspection, weighing various potential outcomes, lifestyle changes, and emotional responses. Introspective participants often voiced thoughtful considerations about pain, diet, immediate postoperative functional limitations, and less commonly, the potential long‐term impacts of surgery. More commonly, patients appeared to engage passively, expressing vague or superficial expectations, or acknowledging they had not given the recovery process much thought at all. When a 70‐year‐old‐male with gallbladder adenocarcinoma was asked if the surgeon shared any information that was helpful in planning for and thinking about surgery, he responded, *'No. It was straightforward. Straightforward knowledge. You know what I mean? Common knowledge'* (P19). In some cases, patients openly stated they had avoided thinking about recovery or deliberately deferred contemplation to *'go with the flow'* (P16). For example, one patient shared *'I just decided I was going to put one foot in front of the other and ignore what surgery was going to be all about…. I dealt with it by ignoring the details. It was a goal'* (P5). This variability reflected a broader spectrum of how patients psychologically approached the uncertainty of surgery and its aftermath.

#### 3.2.3 Expectations Regarding Life Expectancy and Cancer Prognosis

Expectations regarding cancer prognosis and the likelihood of cure also spanned a range of certainty and degrees of contemplation. The majority of patients were uncertain about how surgery was anticipated to affect their likelihood of cure and their overall prognosis. Despite this, many held expectations that they would go on to live a normal lifespan. Many patients had not considered or asked how surgery was expected to impact their overall prognosis and instead remained focused on '*getting [the cancer] out*' (P14). A 40‐year‐old female with PDAC shared, *'I kept telling them, “Just get it out. Come on, just get it done,” again, because I did not realise at the time what I was going through, even though they tried to sit down and they did and explained a lot of things'* (P1). The 78‐year‐old male with PNET shared, *'I guess, I really hadn't thought about that context that the surgery will allow me to live X number of years longer than I would've lived if I didn't have the surgery. I'm expecting to live another 20 years. My mother died when she was 96. I think I can get to that too'* (P23). When asked about any worries or concerns regarding the long‐term effects of surgery and/or the experience of surgical recovery, a 41‐year‐old female with metastatic colon cancer shared, *'I just kind of want to be alive as long as possible… I don't really care that much [regarding what is involved to recover from surgery]'* (P6). Another 41‐year‐old female with goblet cell adenocarcinoma similarly noted, *'I don't want to think that there's a timeline of my life… I'd rather like to think that the surgery got it all… I just want to stay in my happy bubble'* (P13).

#### 3.2.4 Conflicting, Evolving, and Internally Inconsistent Expectations

Patients’ expectations often fluctuated during interviews and contained contrasting thoughts and feelings. Many participants expressed both hopes and fears, confidence and doubt, often within the same interview or even the same sentence. These shifting narratives revealed the emotional and cognitive complexity of preparing for major surgery in the context of cancer. Some patients initially articulated strong expectations of recovery or cure, but later qualified those statements with uncertainty or acknowledged the possibility of complications. Others oscillated between optimism and fatalism, reflecting the difficulty of holding competing emotional truths at once. In some cases, patients appeared to contradict themselves outright, for example, stating that they were not worried and expected surgical recovery to be straightforward while simultaneously describing concern and trepidation. For example, a 41‐year‐old female with colon cancer noted, *'I'm hoping that after 3 to 6 months that eventually I'll incrementally get back to normal… There's always the fear that I'm going to die on the table'* (P6). Similarly, a 40‐year‐old female with pancreatic cancer noted, *'My concern is really that maybe the universe decides, hey, it's time for her to shut down… but I think after a couple of days I might be fine just with Tylenol'* (P1).

#### 3.2.5 Conflation of Hopes With Expectations

Patients often blurred the line between what they hoped for and what they expected, and often did not differentiate between the two. Across all question types related to expectations, including broad prompts such as *'What do you expect recovery to be like?'* and *'Can you tell me about your expectations from surgery?'* to the more specific questions like, '*How do you expect surgery to affect your appetite?'*, patients repeatedly framed their responses in terms of their hopes. For example, *'My hopes are that I will recover really good and that I'll be out and running after 7 days'* (P1) and *'I hope it's going to go quickly good, and that I don't have to throw up a lot, and that I don't feel nauseous and definitely no pain'* (P1). This occurred frequently enough in the first 10 interviews that the interview guide was revised to include a direct follow‐up question inquiring about the difference between expectations and hopes. It became clear that, upon direct questioning that most patients had a unified conceptual understanding of hopes and expectations. Their hopes were their expectations, and they were unable to differentiate between the two. A 61‐year‐old female with ampullary adenocarcinoma, and many others, said *'I guess, my hope and my expectation were the same'* (P11). See Table 4 for additional examples.

### 3.3 Theme 3: Contrasting Anticipated and Actual Recovery After Surgery

Among the group of postoperative participants (*n* = 16; 47%), patients commonly reported experiences that did not align with their preoperative expectations. Though some patients indicated that their recovery was consistent with their anticipations, many encountered unanticipated challenges, from physical symptoms to emotional and practical adjustments that they had not foreseen before surgery. These discrepancies shaped surgical experiences, influenced well‐being, and deepened patients’ understanding of what it meant to have high‐risk surgery for cancer.

#### 3.3.1 The Discordance Between Expectations and the Experience of Surgical Recovery

Surgery unfolded exactly as expected for only a very small number of patients. While some found the experience easier than anticipated, the more common narrative was that surgery was more difficult than expected, even when the postoperative course was medically uncomplicated. Patients who described an easier‐than‐expected recovery often had prior experience with similar procedures or had deliberately prepared themselves. A 47‐year‐old male with rectal cancer and liver metastasis who had undergone liver resection shared, *'I would say it was easier, I think it's more because I've been there, done that, and was prepared for what was going to happen when I woke up'* (P22).

For patients whose experiences were more challenging than they anticipated, the degree of discordance ranged from small to large. Some described relatively minor deviations from expectations, such as slower‐than‐anticipated healing, a 64‐year‐old male with PDAC noted *'I kind of thought I would heal up a little faster…'* (P2) or discomfort that had not been mentioned preoperatively, such as nausea and bloating, *'…I don't believe I was ever informed that you're going to go through some gas pains'* (P2). Others encountered more disruptive postoperative changes than anticipated, particularly related to oral intake and bowel function. A 78‐year‐old female described how, even six and a half weeks after surgery that life still wasn't back to normal. She described, *'It hurts to eat, I can't eat much. I'm repulsed by food*' (P5). Similarly, a 79‐year‐old female shared *'I didn't know I was going to have this diarrhoea problem at all'* (P4).

Several participants described more substantial and distressing mismatches between what they had been told and what they experienced. These included prolonged time with drains, unexpected difficulty urinating without a catheter over a month after surgery, and a recovery that felt far more arduous than the information materials had led them to believe. As a 78‐year‐old female with PDAC described, *'In the [institutional educational] video, there's this cheery little nurse… she makes it sound like you're going to be a little sick for 2 or 3 days, but then you'll feel better, but it's not like that at all'* (P5). Reflecting on the severity of the postoperative pain she shared, *'I didn't know I was going to be in uncontrolled pain for 36 h, screaming. I was told we can control the pain. Well, you can't control the pain because you can't'* (P5).

#### 3.3.2 The Limits of Preoperative Understanding

Several patients commented on the inability to understand what surgical recovery was like until they experienced it for themselves. This sentiment was shared by many and is captured well by a 59‐year‐old female who had undergone a distal pancreatectomy and splenectomy described waking up with a nasogastric tube, drains, and a catheter after surgery: *'I think somebody could have told me. But it probably still wouldn't have hit me until I woke up with it… Somebody can tell you about it, but it's hard to visualise. And it's a lot of information anyway. All the information about the surgery and how things are going to go, and what to expect, and then you listen and you hear and you try to take in as much as possible, but you can't really visualise it. Until it happens'* (P18). A 54‐year‐old patient who had undergone a low anterior resection with radical cystectomy, nephrectomy, resection of multifocal sarcoma and diverting ostomy described learning how to take care of himself at home after discharge, *'I had to learn by experience rather than knowing beforehand'* (P3). Finally, a 78‐year‐old female with PDAC keenly noted, *'There was a lot of areas where I didn't feel at the end of it that I'd been told the truth… At some point, I gave up expectations, because every expectation I had just didn't happen. It was clear to me pretty early on that one of the problems with this is that there aren't averages'* (P5). Additional quotations are captured in Table 5.

**Table 5:** Anticipated versus actual recovery after surgery.

Easier than expected	I thought I was going to be worse off. I thought that I was going to be just limited to the chair and not be able to get up and pick something little to eat. (P13)
	Actually the book made me think that it was going to be harder than it was. It wasn't as‐‐ I was expecting this experience to be worse. (P13)
	It was exactly the same, if not, in my opinion, I would say it was easier… I think it's more because I've been there, done that, and was prepared for what was gonna happen when I woke up. (P22)
	It wasn't as painful as I thought it was gonna be. Don't get me wrong, it was painful, but it wasn't painful as I thought it would be. It was painful, but not in the things I thought it would be. (P33)
	I don't know if it's just that I was—I knew what to expect or if it truly has gone easier this time, but the—yeah, the recovery was a pleasant surprise. (P22)
Harder than expected	They make it sound like it's a little walk in the park and it's not a walk in the park. (P5)
	I had no clue how bad I was going to feel. (P18)
	I didn't realise how uncomfortable it was going to be for me. (P14)
	I expected to just wake up and be like— high‐five the doc, like 'Yeah, we did it!' (P14)
	I was not expecting to be in and out like that, and I was not expecting things to be so foggy. (P14)
	The whole physical thing has been much harder than I've expected. (P5)
	I got to say, this is the most humbling thing that I've ever been through, to not be able to, initially, take care of myself was—I couldn't believe it. (P14)
	I was not expecting to be totally out of it or at the point where I couldn't get up out of bed to go to the bathroom. I'd have to still have the catheter in for a few days. I wasn't expecting things like that. (P18)
	I didn't realise how worn out daily living things were going to make me. (P18)
	I didn't know I was going to have this diarrhoea problem at all. (P4)
	I wasn't expecting [the drains] to affect me the way they did. I don't know how to explain it. I was really sad about it, about having them in me. I thought that they would be out [by the time I left the hospital. (P14)
	I didn't realise what it was going to be like. I just knew that I'd probably be in pain and couldn't do a lot of things after I got home. (P4)
	I'm hoping that after 3 to 6 months that eventually I'll incrementally get back to normal. Hopefully, that's the dream. (P6)
	At some point in this, I gave up expectations, because every expectation I had just didn't happen. It was clear to me pretty early on that one of the problems with this is that there aren't averages. (P5)
	I was thinking, it's just like having a baby. You think you know what it's going to be like, but then, once you're in, you're like, ‘Oh. My gosh.’ (P20)

## 4 Discussion

This qualitative study used a CTA interviewing approach to explore how patients with cancer form expectations about high‐risk abdominal surgery and how those expectations shape preoperative preparation and postoperative recovery. Participants in our study described a range of factors that shaped the development of their expectations and influenced their experience. Patients demonstrated wide variation in the certainty of their expectations, the extent to which they had considered expectations before surgery, and their assumptions about returning to 'normal life'. In the preoperative setting, the inconsistency of patient expectations suggests that they are not static beliefs, but dynamic, context‐sensitive expressions shaped by personal experience, differing sources of information, evolving emotions, and individualised coping strategies. Notably, patients frequently conflated hopes with expectations and often had difficulty differentiating desired outcomes from those they realistically anticipated. Consequently, expectations were often anchored in hope rather than perceived likelihood. This may reflect a psychologically protective tendency to anticipate favourable outcomes in the face of high risk and uncertainty. In this context, expectations may serve not only as predictions about future outcomes but also as mechanisms for maintaining optimism, coping with uncertainty, and preserving a sense of agency during a highly stressful period. Among postoperative participants, experiences of recovery consistently differed from their initial expectations, highlighting gaps in preparation, understanding, and communication about the recovery process. In this context, the emotional burden associated with contemplating serious complications, prolonged recovery, or disease progression might have also influenced how expectations were formed and expressed. Lastly, although not examined systematically in this study, several participants described relying on family members or caregivers during surgical consultations and recovery, suggesting that social support may influence how information is received, interpreted, and retained.

Our findings suggest that patient expectations regarding surgery are not always well‐formed or readily articulable. Expectations appeared to be shaped by multiple sources of information, including conversations with clinicians, prior personal experiences, family and social networks, and information obtained independently. The integration of these diverse and sometimes contradictory influences may help explain why patients frequently expressed expectations that were incomplete, evolving, or internally inconsistent. In the context of high‐risk cancer surgery, these findings suggest that clinicians may need to move beyond simply eliciting expectations toward actively supporting their development and clarification. This distinction between eliciting expectations and helping patients formulate them is particularly relevant to the Ideas, Concerns, and Expectations (ICE) framework, which emphasises the importance of eliciting patient expectations during clinical encounters [34, 35]. Our findings suggest that, while elicitation is necessary, it may be insufficient without additional support to help patients develop, refine, and contextualise their expectations.

The findings from this study align with previous research outlining the need for patient‐centred preoperative support and education for surgical patients undergoing major surgery [8, 14, 36, 37, 38, 39, 40, 41]. For example, in their qualitative work exploring challenges faced by adults undergoing major elective procedures, Keny et al. reported that patients were often surprised by the intensity and duration of postoperative symptoms [38]. These findings have been echoed across multiple studies documenting a persistent mismatch between patients’ expectations and the lived reality of surgical recovery [6, 40, 42, 43, 44, 45, 46, 47, 48, 49, 50]. Such work reinforces the variability in information preferences as well as current gaps in preparation and communication, which not only shape postoperative experiences but may additionally contribute to distress and suffering. The present study extends this literature by illuminating how these gaps emerge specifically in the context of high‐risk abdominal cancer surgery and by describing the cognitive and emotional processes through which patients form and frame their expectations.

There are three key implications from our findings that can inform the development of supportive interventions to improve expectation management for patients undergoing high‐risk abdominal cancer surgery. First, expectations were often underdeveloped, unexamined, or based on incomplete information, suggesting a need for structured preoperative conversations that explicitly elicit what patients believe surgery and recovery will entail. Interventions that strengthen surgeons’ ability to elicit, clarify, and address patients’ expectations may also enhance informed decision‐making, facilitate alignment between operative plans and patient priorities, and help surgeons identify gaps or misconceptions early. Second, patients frequently conflated hopes with expectations, highlighting the importance of helping patients differentiate between desired outcomes and realistic postoperative trajectories. Tools or conversational frameworks that support this distinction while preserving patients’ coping strategies may improve clarity, reduce emotional burden, and foster well‐informed decision‐making. Third, postoperative experiences often diverged significantly from preoperative expectations, indicating opportunities to enhance anticipatory guidance and normalise the variability of recovery. Reinforcing what the early postoperative period may realistically look like, while emphasising uncertainty as an expected part of the process, could reduce distress, strengthen preparedness, and improve the patient experience. These findings also raise important questions regarding the role of health literacy in shaping how patients access, interpret, and apply information when forming expectations, particularly in the context of complex, high‐risk surgical care [51, 52, 53].

### 4.1 Limitations

Participants were recruited through clinician‐led outreach by a single study‐team member based on operative schedules, which may introduce selection bias, as individuals who were reachable and willing to participate may differ from those who declined or did not respond. Second, all interviews were conducted by a single interviewer, which may influence how questions were posed and how participants responded, despite efforts to enhance rigour through iterative guide refinement, analytic memoing, and team‐based coding. Third, participants were recruited from two academic medical centres, which may limit transferability of findings to other clinical settings or populations. Of note, although English was not the primary language of all participants, all participants were able to complete interviews in English. Consequently, the perspectives of patients with limited English proficiency may be underrepresented, and the findings may not fully reflect the communication challenges and expectation‐formation processes experienced by these populations. Fourth, postoperative interviews were conducted across a range of time points following surgery, which may have influenced participant recall and interpretation of their experiences. While this variability allowed for the capture of diverse recovery trajectories, it may introduce recall bias and shape how participants reconstructed their expectations over time. Finally, as with all qualitative research, these findings are not intended to be statistically generalisable, but rather to provide in‐depth insight into patient experiences and perspectives that may inform future research and clinical practice.

## 5 Conclusion

This study provides a deeper understanding of how patients with cancer develop expectations for high‐risk abdominal surgery and how these expectations shape preparation for, and the lived experience of, recovery. Patients drew on diverse and often insufficient sources of information, leading to wide variation in certainty, contemplation, and projected future well‐being. Many conflated hopes with expectations, lived with discordant expectations, and amongst those who had undergone surgery, most experienced some degree of mismatch between what they anticipated and what recovery ultimately entailed. Taken together, these findings underscore the need for more intentional, patient‐centred communication and expectation‐setting in surgical oncology. Future work should evaluate strategies and interventions that facilitate expectation‐aligned counselling and enhance communication for patients undergoing high‐risk surgery for cancer.

## Author Contributions

**Kimberly E. Kopecky:** conceptualization, investigation, funding acquisition, writing – original draft, methodology, validation, visualization, writing – review and editing, formal analysis, supervision. **Jacquelyn E. F. Speer:** writing – review and editing, formal analysis. **Janet Julson:** formal analysis, writing – review and editing. **Alizeh Abbas:** formal analysis, writing – review and editing. **Olivia Monton:** writing – review and editing, methodology, conceptualization. **Fabian M. Johnston:** supervision. **J. Nicholas Odom:** supervision, funding acquisition, writing – review and editing.

## Ethics Statement

This study was deemed exempt by the institutional review boards of The Johns Hopkins Hospital (IRB00399721) and the University of Alabama at Birmingham (IRB‐300012566).

## Conflicts of Interest

The authors declare no conflicts of interest.

## Use of Artificial Intelligence

AI tools were used only for minor editorial support, including grammatical refinement and word choice. No AI tools were used for data analysis, interpretation of findings, or generation of substantive content.

## Supporting information


Supporting File 1

Supporting File 2

## Data Availability

The data that support the findings of this study are available on request from the corresponding author. The data are not publicly available due to privacy or ethical restrictions. Due to the risks of participant identification associated even with deidentified transcripts from patient interviews, full transcripts will not be shared. Summaries of analytic codebooks or analytic memos are available upon request.
