# Supplementary material for: Patient Expectations in High‐Risk Abdominal Surgery for Cancer
Source: Health Expect. 2026 Jul 12;29(4):e70764. doi: 10.1111/hex.70764 (PMC13356890; doi:10.1111/hex.70764)
Supplement: Supplementary file 1 — Supporting File 1 [file HEX-29-e70764-s002.docx]

**Supplementary Table A. Table of Participant Demographics**

| **Participant ID** | **Site** | **Interview Type** | **Age** | **Sex** | **Race** | **Diagnosis** | **Scheduled Procedure** | **Robotic** |
| --- | --- | --- | --- | --- | --- | --- | --- | --- |
| P1 | JHH | Pre-op | 40 | F | W | PDAC | Pancreaticoduodenectomy | N |
| P2 | JHH | Post-op | 64 | M | W | PDAC | Pancreaticoduodenectomy | N |
| P3 | JHH | Post-op | 54 | M | W | Retroperitoneal Sarcoma | Sarcoma resection | N |
| P4 | JHH | Post-op | 79 | F | W | PDAC | Pancreaticoduodenectomy | N |
| P5 | JHH | Post-op | 78 | F | W | PDAC | Pancreaticoduodenectomy | N |
| P6 | JHH | Pre-op | 41 | F | W | Colorectal | CRS/HIPEC | N |
| P7 | JHH | Pre-op | 41 | F | W | PDAC | Pancreaticoduodenectomy | Y |
| P8 | JHH | Pre-op | 75 | M | AAW | Colorectal | Colectomy, hepatectomy, liver Ablation | N |
| P9 | JHH | Post-op | 59 | M | A | Colorectal | CRS/HIPEC | N |
| P10 | JHH | Post-op | 61 | F | W | Retroperitoneal Sarcoma | Sarcoma resection | N |
| P11 | JHH | Pre-op | 61 | F | B | Ampullary adenocarcinoma | Pancreaticoduodenectomy | Y |
| P12 | JHH | Pre-op | 31 | M | W | Cholangiocarcinoma | Hepatectomy, hepaticojejunostomy | N |
| P13 | JHH | Post-op | 41 | F | W | Goblet cell adenocarcinoma | CRS/HIPEC | N |
| P14 | JHH | Post-op | 36 | M | W | Appendiceal mucinous neoplasm | CRS/HIPEC | N |
| P15 | JHH | Pre-op | 63 | F | A | PDAC | Pancreaticoduodenectomy | Y |
| P16 | JHH | Pre-op | 54 | F | B | Colorectal | Colectomy, hepatectomy | Y |
| P17 | JHH | Pre-op | 40 | M | A | Appendiceal adenocarcinoma | CRS/HIPEC | N |
| P18 | JHH | Post-op | 59 | F | W | PDAC | Distal Pancreatectomy, Splenectomy | N |
| P19 | UAB | Post-op | 70 | M | B | Gallbladder adenocarcinoma | Partial hepatectomy, duodenectomy | N |
| P20 | UAB | Post-op | 55 | F | W | Colorectal | CRS/HIPEC | N |
| P21 | UAB | Post-op | 58 | M | B | PNET | Pancreaticoduodenectomy | N |
| P22 | UAB | Post-op | 47 | M | -- | Colorectal | Hepatectomy | N |
| P23 | UAB | Pre-op | 78 | M | B | PNET | Distal Pancreatectomy | Y |
| P24 | UAB | Pre-op | 77 | M | W | Hepatocellular carcinoma | Hepatectomy, Hepaticojejunostomy | N |
| P25 | UAB | Pre-op | 60 | F | W | PDAC | Pancreaticoduodenectomy | N |
| P26 | UAB | Post-op | 65 | F | B | PDAC | Distal pancreatectomy, en-bloc celiac axis resection and splenectomy | N |
| P27 | UAB | Pre-op | 67 | M | W | PDAC | Distal Pancreatectomy, splenectomy | Y |
| P28 | UAB | Pre-op | 85 | F | W | Colorectal | Hemicolectomy | N |
| P29 | UAB | Pre-op | 42 | F | W | Anal SCC | Abdominoperineal resection | N |
| P30 | UAB | Pre-op | 75 | M | -- | PDAC | Distal Pancreatectomy | Y |
| P31 | UAB | Post-op | 58 | F | B | NET | Neuroendocrine tumor of small bowel | N |
| P32 | UAB | Pre-op | 77 | F | B | Gastric GIST | Partial gastrectomy | Y |
| P33 | UAB | Post-op | 60 | M | B | Colorectal | Abdominoperineal resection | N |
| P34 | UAB | Pre-op | 41 | M | W | Colorectal | Hepatectomy, ablation | N |

**JHH**- **UAB**-; **F**- Female; **M**- Male; **W**- White; **B**- Black; **A**-Asian, **AAW**- African American White; **PDAC**- Pancreatic ductal adenocarcinoma, **PNET-** Pancreatic neuroendocrine tumor; **SCC**- Squamous cell carcinoma; **GIST**- Gastrointestinal stromal tumor; **CRS/HIPEC**- cytoreductive surgery with hyperthermic intraperitoneal chemotherapy; **Y**- Yes; **N**- No
